# Effect of percutaneous stenting strategy of unresectable malignant hilar biliary obstruction by three‐dimensional reconstruction volumetry

**DOI:** 10.1002/cam4.5720

**Published:** 2023-02-20

**Authors:** Xiaobo Fu, Weiwei Jiang, Maoyuan Mu, Guobao Wang, Han Qi, Zixiong Chen, Mengxuan Zuo, Fei Gao

**Affiliations:** ^1^ Department of Minimally Invasive & Interventional Radiology Sun Yat‐sen University Cancer Center, State Key Laboratory of Oncology in South China, Collaborative Innovation Center for Cancer Medicine Guangzhou China; ^2^ Department of Endoscopy Sun Yat‐sen University Cancer Center, State Key Laboratory of Oncology in South China, Collaborative Innovation Center for Cancer Medicine Guangzhou China

**Keywords:** liver volume, malignant hilar biliary obstruction, predictors, stent implantation, survival

## Abstract

**Purpose:**

To explore clinical outcomes of percutaneous stent implantation using volumetric criteria for unresectable malignant hilar biliary obstruction (MHBO). Additionally, aimed to identify the predictors of patients' survival.

**Methods:**

Seventy‐two patients who were initially diagnosed with MHBO between January 2013 to December 2019 in our center were retrospectively included. Patients were stratified according to the drainage achieved ≥50%, <50% of the total liver volume. Patients were divided into two groups: Group A (≥50% drainage), and Group B (<50% drainage). The main outcomes were evaluated in terms of relief of jaundice, effective drainage rate, and survival. Related factors that affect survival were analyzed.

**Results:**

62.5% of the included patients reached effective biliary drainage. The successful drainage rate was significantly higher in Group B than in Group A (*p* < 0.001). The median overall survival (mOS) of included patients was 6.4 months. Patients who received drainage ≥50% of hepatic volume achieved longer mOS than those who received drainage <50% of hepatic volume (7.6 months vs. 3.9 months, respectively, *p* = 0. 011). Patients who received effective biliary drainage had longer mOS than those who received ineffective biliary drainage (10.8 months vs. 4.4 months, respectively, *p* < 0.001). Patients who received anticancer treatment had longer mOS than those who only received palliative therapy (8.7 months vs. 4.6 months, respectively, *p* = 0.014). In the multivariate analysis, KPS Score ≥ 80 (*p* = 0.037), ≥50% drainage achieved (*p* = 0.038), and effective biliary drainage (*p* = 0.036) were protective prognostic factors that affected patients' survival.

**Conclusion:**

Drainage achieved ≥50% of the total liver volume by percutaneous transhepatic biliary stenting seemed to have a higher effective drainage rate in MHBO patients. Effective biliary drainage may create chances for these patients to receive anticancer therapies that seem to provide survival benefits.

## 1 INTRODUCTION

Malignant hilar biliary obstruction (MHBO) means stenosis or blockage of the biliary tract due to tumor infiltration or external compression.^1^, ^2^ Various types of malignancies can lead to biliary obstruction, such as cholangiocarcinoma (CCA), gallbladder cancer; liver cancer, pancreatic cancer, and metastasis to lymph nodes around the bile duct.^3^ MHBO patients are frequently detected at an advanced stage, 80% of the MHBO cases are unresectable.^1^, ^4^

Stents implantation is widely recommended as the preferred palliative modality for unresectable MHBO cases.^5^, ^6^, ^7^ Biliary drainage through stent can ameliorate symptoms of jaundice, preserve deteriorated liver function, and improve quality of life. Furthermore, it may create chances for these patients to receive anticancer therapies. Commonly, anticancer therapies include chemotherapy, molecular target drugs, and immune checkpoint inhibitors, which usually require good liver function.^8^, ^9^, ^10^, ^11^ Thus, successful biliary drainage plays a vital role in creating chances for unresectable cancer patients with MHBO to receive the above anticancer treatments.

The endoscopic approach is frequently the first choice to perform biliary drainage compared to surgery or percutaneous approaches,^5^, ^12^ but for hilar obstruction, a recent randomized controlled trial^13^ failed to compare percutaneous drainage and endoscopic drainage as the primary intervention in patients with MHBO. Although the preferred procedure remains uncertain,^14^ percutaneous transhepatic biliary stenting (PTBS) seems a valuable and safe technique in the management of hilar biliary obstruction, which has already been proved in observational data.^15^, ^16^, ^17^, ^18^, ^19^ Selectively targeted drainage aimed at decompressing at least 50% of the viable liver volume may obtain improved drainage outcomes.^5^ Frequently, planned drainage was based on MRI/CT images with the goal of adequate numbers of sectors to decompress more than 50% of the viable liver volume.^20^ The above strategy has only been evaluated by the endoscopic approach.^21^, ^22^, ^23^ Few research studies are available on percutaneous stenting.^24^ Thus, the current retrospective study aimed to compare the drainage and survival outcomes of different volumetric criteria by the percutaneous approach for malignant hilar stricture caused by various cancer. In addition, there is no previous study to assess the efficacy of biliary stenting with liver drainage volume using three‐dimensional (3D) reconstruction. Furthermore, this study explored the predictors of patients' survival.

## 2 MATERIALS AND METHODS

This retrospective study was approved (approval number: SL‐B2022612‐01) by the Institutional Review Board of Sun Yat‐sen University Cancer Center, which waived the need for written informed consent and conformed to the Declaration of Helsinki of the World Medical Association (2013). We retrospectively reviewed the medical records of 115 patients with MHBO at the time of unresectable cancer diagnosis who received biliary stent implantation at the center from January 1, 2013 to December 31, 2019.

**FIGURE 1:**
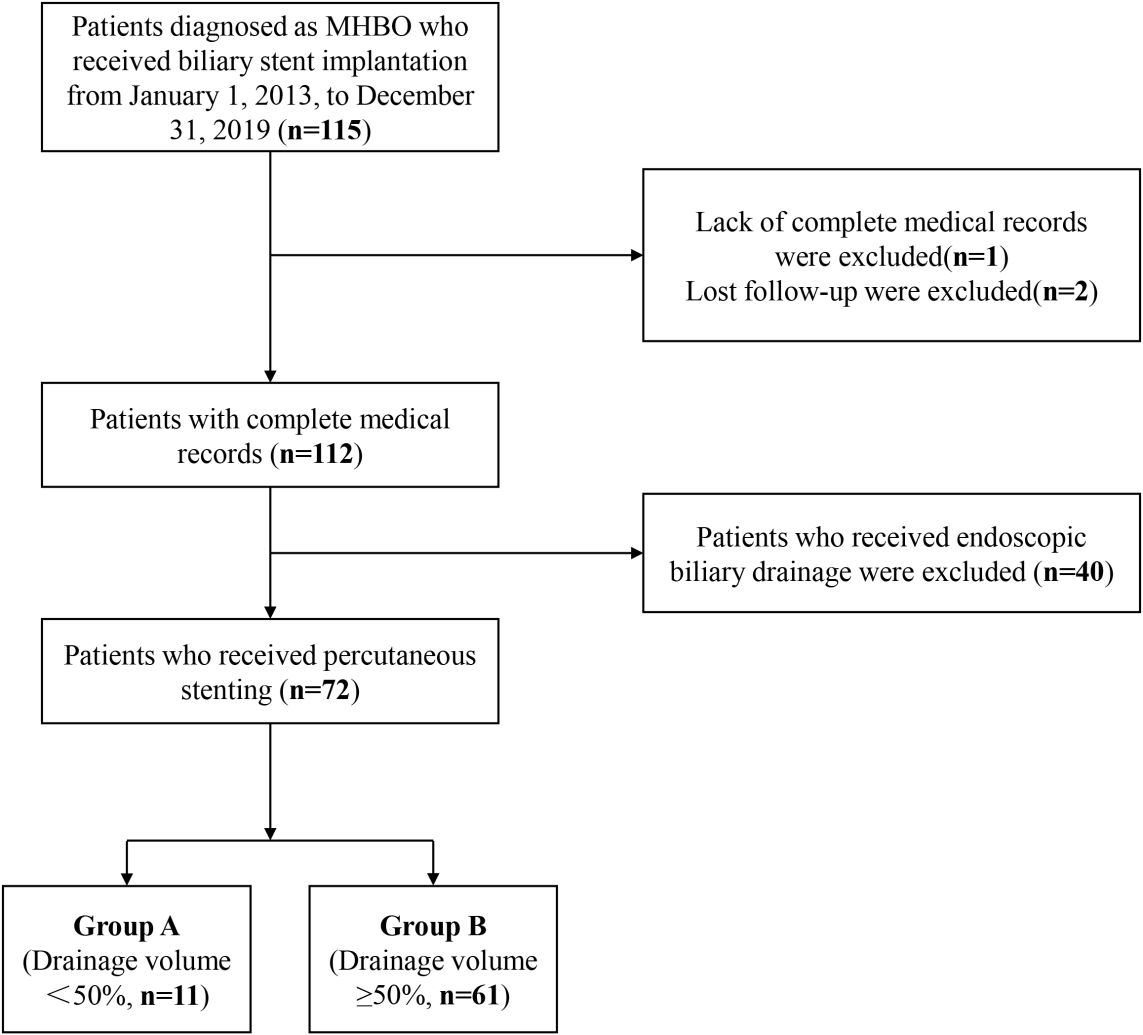
Patients flow diagram.

Inclusion criteria: (1) unresectable malignancies evaluated by two senior surgeons with experience of more than 10 years or patient's unwillingness to receive surgical resection; (2) a confirmed MHBO (Bismuth–Corlette type II–IV) diagnosis based on laboratory, pathological or radiologic evidence; (3) no previous biliary interventional treatment before admission; (4) patients who received biliary stent implantation. Exclusion criteria: (1) patients who received endoscopic biliary drainage; (2) incomplete medical records; (3) lost follow‐up. Forty‐three patients were eliminated from the study: received endoscopic biliary drainage (*n* = 40); incomplete medical record (*n* = 1); lost follow‐up (*n* = 2). Eventually, 72 patients were included in this study. (Figure 1).

### 2.1 The type of Bismuth

The type of MHBO was based on radiological images (enhanced multidetector‐row CT, enhanced MRI, MRCP, or direct cholangiography) by two independent experienced senior radiologists. The Bismuth–Corlette^25^ classification system is referred to classifying different hilar malignancies.

### 2.2 Data collection and follow‐up

The medical records of the 72 included patients were reviewed by two independent doctors, including (1) baseline parameters of patients; (2) laboratory tests examinations; (3) tumor type; (4) treatment of malignant obstructive jaundice; (5) treatment of cancer. Follow‐up for survival began with the diagnosis of unresectable cancer with MHBO in the one enrolled patient. The follow‐up visits consisted of routine outpatient and telephone interviews. All patients were followed up regularly until November 30, 2022, or patient death.

### 2.3 Clinical outcomes and definitions

The main clinical outcomes of this study were the drainage outcome and survival outcome. The second outcome was procedure‐related complications. Effective biliary drainage was defined as a decrease in the total bilirubin concentration of more than 50% of the prestenting value or a decrease to <3 mg/dL within 1 month, and no cholangitis.^17^, ^26^ Major complications referred to those that result in death or serious adverse effects including sepsis, uncontrollable biliary or intestinal bleeding, and acute renal failure; others were considered minor complications.

### 2.4 PTBS procedure

A 22‐G Chiba needle (Cook, Bloomington, IN) was used to percutaneously puncture the dilated peripheral intrahepatic bile duct. Then, a Neff percutaneous access set was introduced into the bile duct, and cholangiography was performed through the outer cannula to assess the location and length of the stenotic site. A 0.035‐in., 180‐cm long guidewire was induced to cross the blockage location, and the outer cannula was introduced over the guidewire to cross the stenosis. Then, one or two SEMSs (self‐expanding metallic stents) (Wallstent [Boston Scientific, Natick]) were implanted. Repeat cholangiography was conducted to confirm stent patency. At last, an external drainage catheter (8.5F, Cook) was inserted percutaneously into an appropriate intrahepatic duct simultaneously for a few days. If there were no complications, and satisfactory stent drainage was confirmed by an interventional radiologist, the external catheter was removed.

### 2.5 3D reconstruction and calculation of liver volume

**FIGURE 2:**
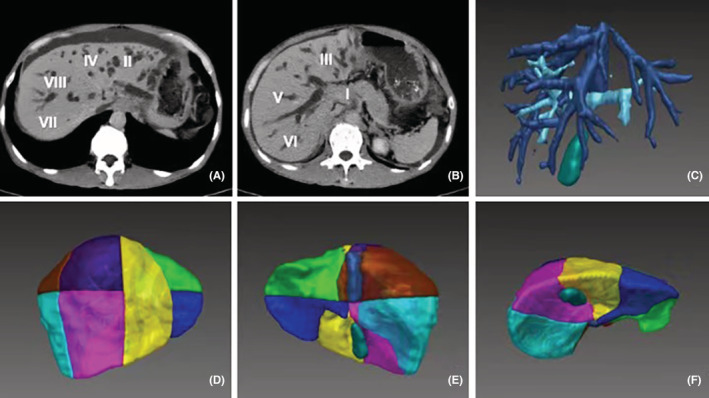
Assessment of drainage volume using 3D modeling. (A,B) Contrast‐enhanced CT scan of MHBO patient. (C) Reconstruction of the hepatic vein and portal vein system. (D,E,F) Ventral, dorsal, and caudal sight of 3D hepatic segments.

Two independent radiologists with 5 years' experience were responsible for delineating the liver and tumor, and a senior radiologist was involved to make the final decision when a major discrepancy occurred. First, 2.5‐mm thick MRI or CT scan slices were transformed into a DICOM format and imported into the Medi‐GPS 3D Visualization System (Demetics Surgical Planning & Navigation System, Demetics Medical Technology, Hangzhou, China). The live region was three‐dimensionally reconstructed by the software. Then, the portal vein and hepatic vein were delineated for the anatomical segmentation of the liver. Three parts of the liver were identified: the right anterior lobe (segments V and VIII), right posterior lobe (segments VI and VII), and left lobe (segments II and III). The classification of segment IV was determined by the actual anatomy of the biliary system, because of possible drainage to either lobe. The last step delineated the internal tumor, and the liver volume of each lobe excluding tumors was calculated for the study (Figure 2).

### 2.6 Statistical analysis

Continuous variables were expressed as the mean ± standard deviation and medians and ranges for and as the number of subjects (percentages) for categorical variables unless otherwise stated. Student's *t*‐test or Mann–Whitney *U* test was used to analyze differences between groups of continuous variables. Person's chi‐squared and Fisher's exact tests were used to compare categorical variables. Overall survival rates were calculated by Kaplan–Meier survival analysis. The log‐rank test Mantel‐Cox was performed to compare survival among different groups. A Cox regression model was built to confirm the different factors that affect patient survival for the multivariate analysis. Significant statistical difference was considered only when a *p*‐Value was less than 0.05. All statistical analyses were performed by using IBM SPSS for Windows version 26.0 (SPSS, Chicago, IL, USA).

## 3 RESULTS

### 3.1 Patient characteristics

This study included 72 unresectable cancer patients with MHBO (male, 75% [54 of 72]; female, 25% [18 of 72]) with a mean age at the time of admission of 57.3 ± 12.8 years (mean ± standard deviation). The etiology included CCA (*n* = 20), hepatocellular carcinoma (*n* = 15), gallbladder carcinoma (*n* = 4), and metastatic malignancies (*n* = 33). Sixty‐five patients had their diagnoses established by pathologic examination, while the remaining seven patients had their diagnoses determined by radiological and laboratory tests. The obstruction site was the hilar biliary including Bismuth type II (40.2% [29 of 72]), Bismuth type III (31.9% [23 of 72]), and Bismuth type IV (27.8% [20 of 72]), respectively. The median liver volume of the right anterior lobe was 0.38 [0.30–0.45], whereas the volume of the right posterior lobe and left lobe was 0.27 [0.23–0.32], and 0.34 [0.26–0.43], respectively (Table 1).

**TABLE 1:** Patient characteristics.

Parameter	All (*N* = 72)
Gender, male/female	54/18
Age (y)	57.3 ± 12.8
Etiology	
Cholangiocarcinoma	20
Hepatocellular carcinoma	15
Gallbladder carcinoma	4
Metastasis	33
Bismuth–Corlette classification	
II/III/IV	29/23/20
Child–Pugh class	
Stage B/C	68/4
The volume of right lobe	0.67 [0.57–0.74]
The volume of left lobe	0.34 [0.26–0.43]
<50%/50%–75%/75%	12/44/16
Unilateral drainage/bilateral drainage	61/11
TBIL (μmol/L)	
Before PTBS	225.0 [140.3–320.2]
4 weeks after PTBS	93.0 [33.5–188.7]
Complications	31
Anticancer treatment	
Yes/No	44/28

*Note*: All values are expressed as mean ± standard deviation, median [range], or number (%).

Abbreviation: TBIL, total bilirubin.

### 3.2 Drainage outcomes

The fraction of liver volume drained was inferred from the relationship between the sectors intubated during PTBS and the volume of each sector as measured by calculation of liver volume assisted by 3D reconstruction. Patients were stratified according to the drainage achieved ≥50% of the total liver volume (Group A), and <50% of the total liver volume (Group B). Age, gender, Karnofsky Performance Status (KPS) score, Bismuth type, cancer stage, biliary tumor, Child–Pugh class, unilateral/bilateral stenting, and median preprocedural bilirubin values were no statistical difference between the two groups (all *p* > 0.05). Effective biliary drainage was achieved in 62.5% (45/72) of the patients. In Group A (≥50% drainage), the postprocedural TBIL is significantly higher than in Group B (<50% drainage) (*p* = 0.003). In addition, the effective biliary drainage rate was significantly higher than in Group B compared to Group A (*p* < 0.001). No major complications were observed in 72 patients. Both groups' mild abdominal pain that occurred in 27 patients was alleviated after analgesic treatment. Four patients manifested chill, fever, and loss of appetite with transient elevation of neutrophil counts and inflammatory factors after the operation, which all returned to normal upon discharge from the hospital. (Table 2).

**TABLE 2:** Characteristics and outcomes of patients depending on liver volume drained(*N* = 72).

Parameter	Group A (*n* = 11) (drainage volume <50%)	Group B (*n* = 61) (drainage volume ≥50%)	*p*‐Value
Age (y)	60.5 ± 4.0	56.7 ± 1.6	0.378
Gender (male/female)	8 (72.7)/3 (23.3)	46 (75.4)/15 (24.6)	1.000
Cancer stage^a^ (III/IV)	2 (18.2)/9 (81.8)	10 (16.4)/51 (83.6)	1.000
KPS Score ≥80, <80	8 (72.7)/3 (27.3)	26 (43.4)/35 (57.3)	0.101
Anticancer therapy (Yes/No)	5 (45.5)/6 (54.5)	39 (63.9)/22 (36.1)	0.247
Biliary tumor	3 (27.3)	21 (34.4)	0.741
Bismuth II/III/IV	4 (36.4)/3 (27.3)/4 (36.4)	25 (40.1)/20 (32.8)/16 (26.2)	0.841
Child–Pugh B/C	11 (100)/0 (0)	57 (93.4)/4 (9.8)	1.000
Unilateral/bilateral	9 (81.8)/2 (18.2)	52 (85.2)/9 (14.8)	0.672
The right liver lobe	0.66 [0.62–0.88]	0.67 [0.56–0.74]	0.531
TBIL before PTBS	190.0 [106.0–270.0]	227.0 [148.0–353.0]	0.294
Complications	4 (36.4)	27 (44.3)	0.747
Effective drainage	1 (11.1)	44 (72.1)	<0.001*
Post TBIL after 4 weeks (μmol/L)	204.9 [137.2–264.3]	85.2 [30.6–151.6]	0.003*

*Note*: All values are expressed as mean ± standard deviation, median [range], or number (%).

Abbreviations: KPS, karnofsky performance status; PTBS, percutaneous transhepatic biliary stenting; TBIL, total bilirubin.

^a^ American Joint Committee on Cancer (AJCC) TNM system based on type of tumor.

* *p*‐value <0.05 was considered to indicate statistical significance.

### 3.3 Survival outcomes

**FIGURE 3:**
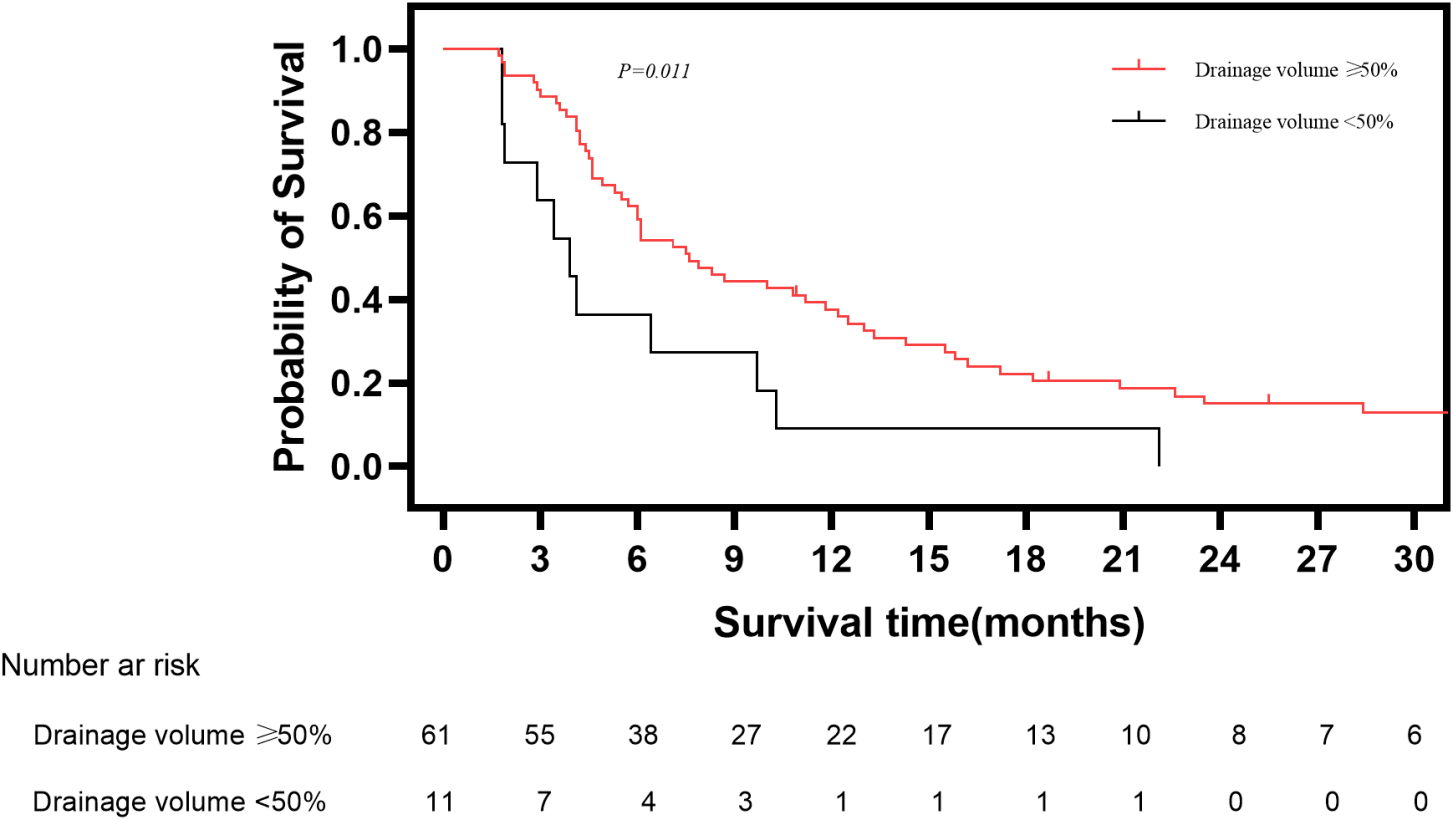
Comparison of overall survival of patients who received ≥50% drainage and patients who received <50% drainage

**FIGURE 4:**
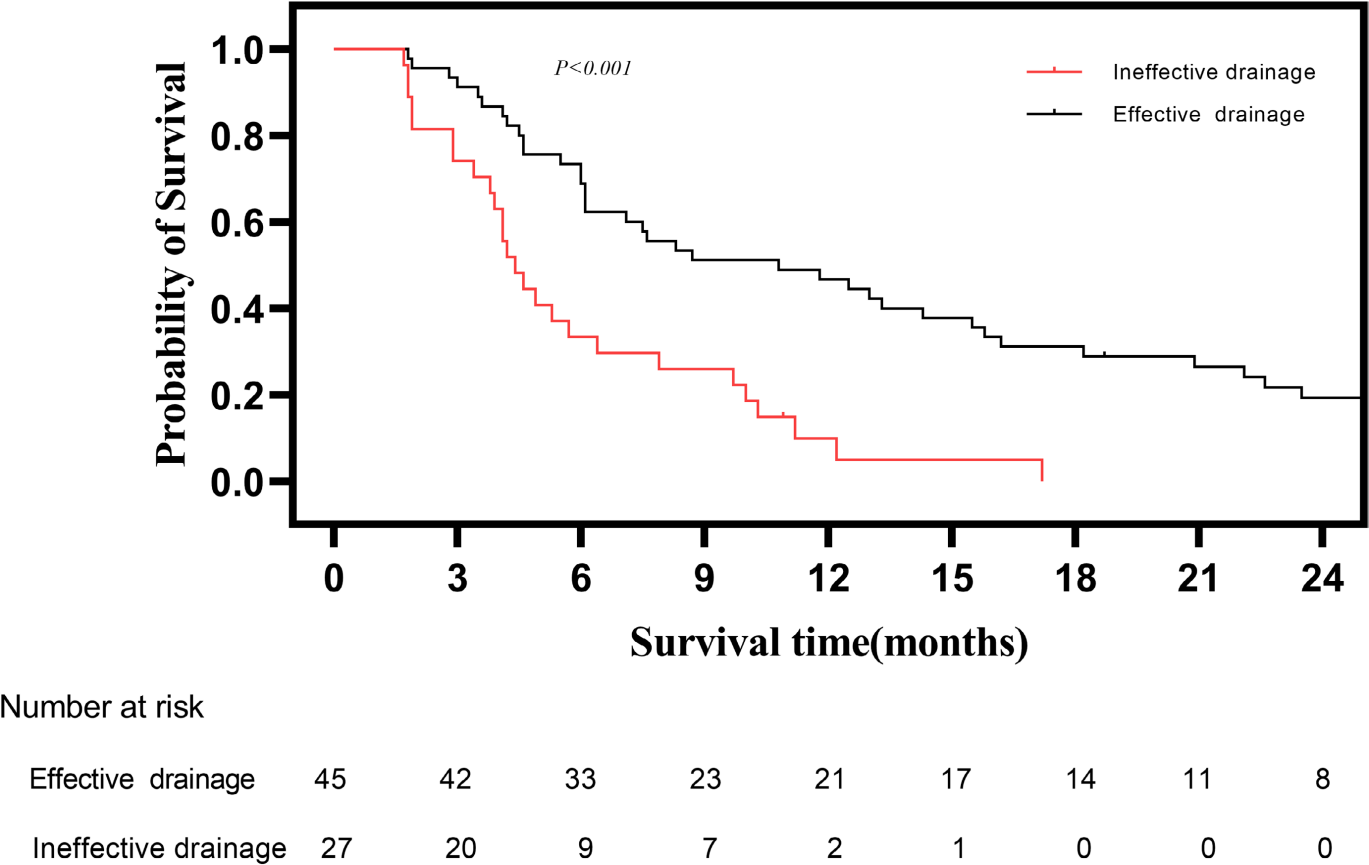
Comparison of overall survival of patients who received effective biliary drainage and patients who received ineffective biliary drainage

**FIGURE 5:**
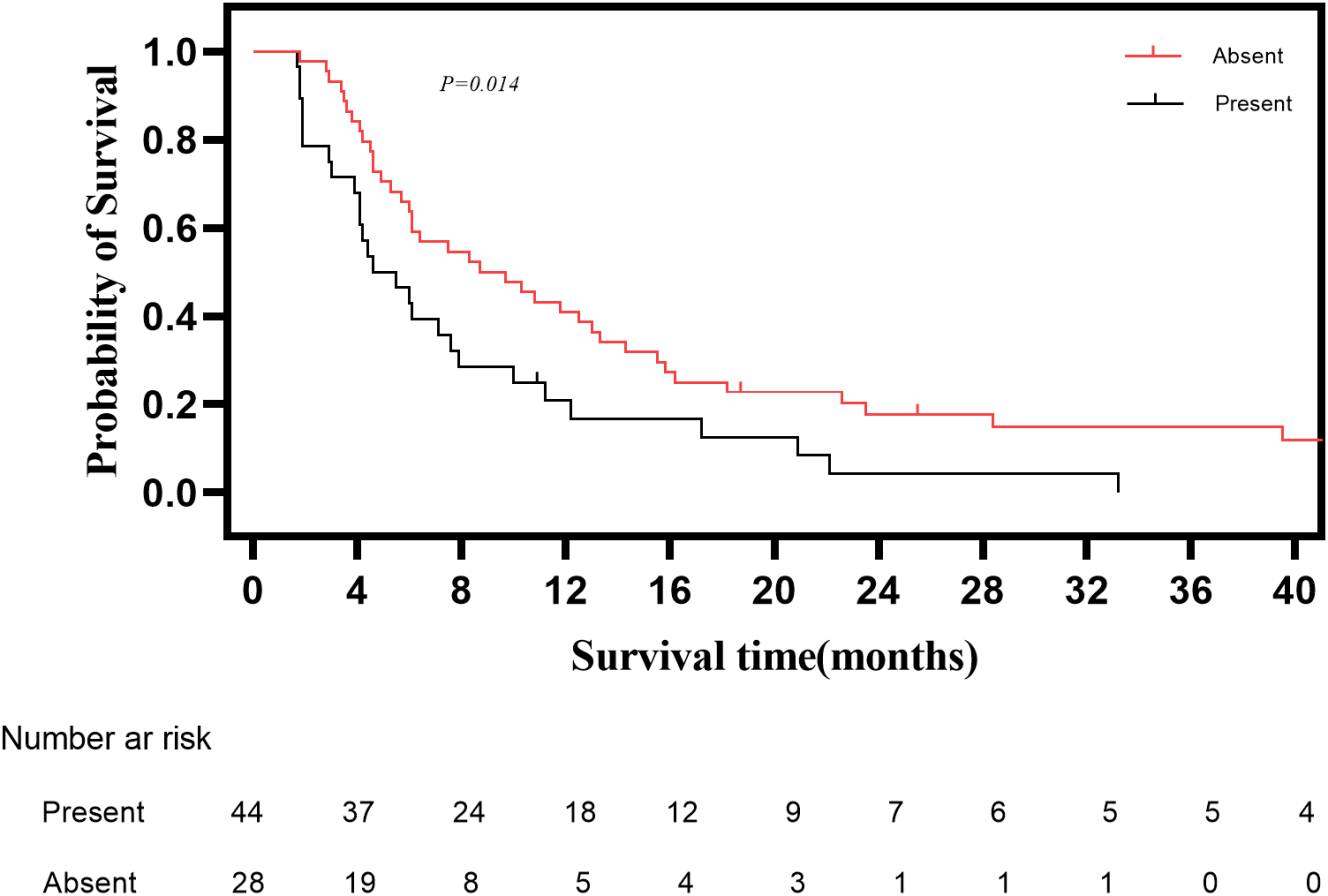
Comparison of overall survival of patients who received sequential anticancer treatment and patients who only received conservative medical treatment

The median follow‐up time was 53.9 months for all patients, median overall survival (mOS) was 6.4 months. The 3‐month, 6‐month, 1‐year, and 3‐year survival rate was 71.8%, 48.0%, 27.1%, and 8.5%, respectively. Patients who received ≥50% drainage achieved longer mOS than those who received <50% drainage (7.6 months vs. 3.9 months, respectively, *p* = 0. 011) (Figure 3). Patients who received effective biliary drainage had longer mOS than those who received ineffective biliary drainage (10.8 months vs. 4.4 months, respectively, *p* < 0.001) (Figure 4). Among 72 included patients, 61.1% (44/72) patients received sequential anticancer treatment after PTBS, and 38.9% (28/72) patients only received conservative medical management. Patients who received anticancer treatment had longer mOS than those who only received palliative therapy (8.7 months vs. 4.6 months, respectively, *p* = 0.014) (Figure 5).

### 3.4 Prognostic factors

In a univariate analysis to determine the prognostic variables affecting the survival of included patients, the Child–Pugh class, KPS score, drainage achieved volume, effective biliary drainage, and anticancer treatment were statistically significant (*p* < 0.05). Ultimately, five items were included in the multivariate analysis. The Child–Pugh class C [hazard ratio (HR) 3.27, *p* = 0.030], KPS score ≥ 80 (HR 0.56, *p* = 0.037), ≥50% drainage (HR 0.45, *p* = 0.038), and effective biliary drainage (HR 0.50, *p* = 0.036) were independent prognostic factors (Table 3).

**TABLE 3:** Multivariate analysis of variables affected the survival of included patients (*N* = 72).

Parameter	Univariate analysis		Multivariate analysis	
	HR (95% CI)	*p*‐Value	HR (95% CI)	*p*‐Value
Sex, male versus female	1.18 (068–2.05)	0.559	–	–
Age, ˃65 versus ≤65 years	1.33 (0.80–2.20)	0.274	–	–
Child–Pugh class, C versus B	2.88 (1.02–8.13)	0.046*	3.27 (1.13–9.53)	0.030*
KPS score ≥80 versus <80	0.55 (0.34–0.91)	0.020*	0.56 (0.33–0.97)	0.037*
Bismuth, III + IV versus II	1.12 (0.67–1.85)	0.669	–	*–*
Biliary tumor	0.75 (0.45–1.26)	0.277	–	*–*
Unilateral drainage/bilateral drainage	1.12 (0.59–2.15)	0.729	–	*–*
Complications, No versus Yes	0.80 (0.49–1.32)	0.383	–	*–*
Drainage achieved ≥50% versus <50%	0.44 (0.23–0.84)	0.014*	0.45 (0.22–0.96)	0.038*
Effective biliary drainage	0.34 (0.20–0.59)	<0.001*	0.50 (0.26–0.96)	0.036*
Treatment to cancer, absent versus present	1.87 (1.13–3.09)	0.015*	1.263 (0.72–2.21)	0.414

Abbreviation: KPS, karnofsky performance status.

* *p*‐Value <0.05 was considered to indicate statistical significance.

## 4 DISCUSSION

Malignancies involving the hilar biliary tract could gradually lead to severe biliary stenosis. Cholestasis occurs in these patients with symptomatic jaundice, which is usually associated with poor prognosis.^2^, ^3^ It can shorten patients' life expectancy due to nutritional disturbance and progression of liver failure.^4^ Furthermore, by affecting the normal excretion of bile, MHBO, which frequently resulted in insufficient liver function could deprive unresectable cancer patients of receiving anticancer therapies.

Ameliorating liver function by relieving jaundice may help to create chances for these patients to receive anticancer treatments and prolong survival. Thus, effective drainage matters, in the current study, the survival of unresectable cancer patients with MHBO who did not receive effective biliary drainage was markedly shorter than those who received effective biliary drainage (4.4 months vs. 10.8 months, respectively, *p* < 0.001). Furthermore, malignant biliary obstruction was a strong risk factor related to patients' survival mentioned in several previous studies.^27^, ^28^, ^29^ Thus, reducing bilirubin and preserving liver function become the main targets of inoperable MHBO cases. Significantly, creating chances for unresectable MHBO patients to receive antitumor treatments, which may help to improve patients' prognosis.^27^, ^30^

In this study, patients who received anticancer treatment achieved a longer survival than those who only received palliative therapy (8.7 months vs. 4.6 months, respectively, *p* = 0.014). Moreover, in the real world, these patients frequently find it difficult to meet the requirements of anticancer therapies due to damage to liver function by biliary obstruction. For example, some of the included patients were hepatocellular carcinoma (HCC) patients. These patients with biliary invasion are usually complicated with symptomatic obstructive jaundice, Although the recommended systemic treatment modalities of HCC, such as atezolizumab plus bevacizumab, tremelimumab plus durvalumab, sorafenib, and lenvatinib demonstrated their valuable advantages in prolonging advanced HCC patients' survival.^8^, ^9^, ^10^, ^11^ However, the above treatments normally require good liver function (mostly Child–Pugh class A). All patients in the current study had Child–Pugh B or C liver function, and patients had difficulty meeting the liver function criteria for anticancer therapy. Thus, ameliorating liver function by relieving jaundice may help to create opportunities for these patients to receive anticancer treatments and prolong survival. Above 60% of the included patients in this study received the following anticancer therapies. Moreover, successful biliary drainage in this study (HR 0.35, *p* < 0.001) was a favorable prognostic factor related to patients' survival. Successful biliary drainage demonstrated a critical role in preparing these patients to receive anticancer therapies, confirmed in a recently published study.^30^

However, controversy remains concerning the importance of bilateral stenting in the palliation of MHBO.^31^, ^32^, ^33^ Recently, the prior focus has moved from “unilateral or bilateral” stenting towards the number of sectors drained based on the amount of viable hepatic volume drained. Because the three liver sectors have substantial variations in the degree of atrophy, congenital junction, and tumor occupation. A single stent sometimes decompresses ≥50% of the viable hepatic volume. while “bilateral stenting” may not, particularly when the left lobe is atrophic, as commonly seen in CCA arising in the left lobe. To some extent, the liver volume of biliary drainage can instruct the selection of either a unilateral or bilateral stent and predict the efficacy of drainage.^20^, ^22^ This concept is fundamentally new regarding biliary drainage from “unilateral” or “bilateral” stent placement, also, current guidelines mentioned drainage ≥50% of the total liver volume is preferred for hilar malignant stricture instead of focusing on the number of stents.^22^, ^23^, ^34^

However, it is not easy work to manage biliary drainage successfully owning that all cases involved a high level of the biliary tract (Bismuth II–IV) in this study, although we knew that a key factor to achieve effective drainage is enough drainage volume. Two‐dimensional CT or MRI scan slices were commonly used to estimate patients' liver drainage volume. However, the precise quantification of hepatic volume is clinically challenging considering the variations of three liver sectors. In this study, we utilized a 3D visualization system for liver segmentation based on the portal/hepatic vein system, which was proven to be more accurate and efficient than the traditional algorithm, especially for livers with low gray or high gray lesions. With the application of a 3D visualization system, the right/left hepatic lobe volume and tumor volume all can be delineated. Thus, it may guide radiologists before the percutaneous stenting in managing hilar biliary stricture caused by various cancers. In this study, we also obtained the same result: the effective biliary drainage rate in the more than 50% drainage class was significantly higher than in the less than 50% group, which is consistent with the previous reports.^22^, ^23^, ^34^

In addition, according to the European guideline,^5^ percutaneous transhepatic biliary drainage (PTBD) or a combination of PTBD and endoscopic retrograde cholangiopancreatography (ERCP) for Bismuth type III and IV cases is recommended. But no clear census on which approach to perform stent implantation is preferred. ERCP with multisegmental drainage is technically difficult owning stricture's tightness, and cannulation of the intrahepatic duct is also highly demanding.^35^ Moreover, during the ERCP procedure, cannulation was preferably attempted using a guidewire through a sphincterotome. But the sphincterotome sometime could be infiltered by malignant masses,^36^, ^37^ the bile duct drainage is not accessible by ERCP. Jang et al reported that the PTBS had higher technical success (100% vs. 72.4%),^38^ which may be due to the percutaneous approach having its advantage in reaching the lobar section. A multiple‐center comparative study by Paik et al.^17^ concluded that in patients with hilar CCA, endoscopic stenting had a lower successful drainage rate compared with the percutaneous approach (77.3% vs. 92.7%, respectively, *p* = 0.049). Moreover, a high‐quality meta‐analysis^39^ that included 546 patients from nine research concluded that PTBS owned a higher effective drainage rate than ERCP in patients with MHBO cases. The above three high‐quality studies proposed the same biliary drainage method of managing hilar biliary obstruction as the current study.

### 4.1 Limitation

Certain limitations should be considered when interpreting this study's results. Potential bias to the work's retrospective nature and the sample size restriction limited the clinical significance. Although our target population was heterogeneous concerning the different histological types of cancer, the mixed cases reflect the actual percutaneous practice. Another limitation of the current study is the method of volume assessment. Draining only 25% of the liver has been accepted to be the minimal requirement for jaundice relief, all cases in this study, the drainage volume was more than 30%, and more than 50% of the drainage volume of viable hepatic volume is recommended to be drained. So, we chose less than 50% as the first volume threshold. It was not accessible in this retrospective research to compare pre‐ and postprocedure CT or MRI scans; This study was conducted based on the relationship between prepercutaneous stenting CT or MRI scans and periprocedural cholangiograms. Studies with larger sample sizes or randomized controlled trials are needed to verify the results.

## 5 CONCLUSIONS

The survival of unresectable cancer patients complicated with MHBO at the initial diagnosis is generally poor. Effective percutaneous biliary drainage seemed to provide survival benefits in these patients with preserved liver function. Drainage achieved ≥50% of the total liver volume by using percutaneous stent implantation seemed to have a higher effective drainage rate in this retrospective study. Effective biliary drainage may create a chance for advanced cancer patients with MHBO to receive anticancer therapies and obtain survival benefits.

## AUTHOR CONTRIBUTIONS

**Xiaobo Fu:** Conceptualization (equal); data curation (equal); formal analysis (equal); investigation (equal); methodology (equal); validation (equal); writing – original draft (lead); writing – review and editing (equal). **Weiwei Jiang:** Formal analysis (equal); investigation (equal); methodology (equal); validation (equal); writing – review and editing (equal). **Maoyuan Mu:** Data curation (equal); formal analysis (equal); writing – review and editing (equal). **Guobao Wang:** Data curation (equal); formal analysis (equal); writing – review and editing (supporting). **Han Qi:** Data curation (equal); formal analysis (equal); writing – review and editing (equal). **Zixiong Chen:** Data curation (equal); writing – review and editing (equal). **Mengxuan Zuo:** Data curation (equal); software (lead); supervision (equal); writing – review and editing (equal). **Fei Gao:** Conceptualization (equal); project administration (lead); supervision (equal); writing – review and editing (equal).

## FUNDING INFORMATION

This study has received funding from the Natural Science Foundation of Guangdong Province (2022A1515010490).

## CONFLICT OF INTEREST STATEMENT

All authors declared that they do not have anything to disclose regarding the conflict of interest concerning this manuscript.

## ETHICS STATEMENT

The Institutional Review Board of Sun Yat‐sen University Cancer Center approved this study. (Document number: SL‐B2022612‐01).

## PATIENTS CONSENT STATEMENT

Written informed consent was waived from all subjects (patients) in this study.

## Data Availability

The datasets used and/or analyzed during the current study are available from the corresponding author upon reasonable request.
